# Potential application value of pigment epithelium-derived factor in sensorineural hearing loss

**DOI:** 10.3389/fnins.2023.1302124

**Published:** 2023-12-14

**Authors:** Zihui Sun, Xiaoguang Li, Guangfei Li, Ying Xu, Jie Meng, Wei Meng, Shuangba He

**Affiliations:** ^1^Department of Otolaryngology Head and Neck Surgery, Nanjing Tongren Hospital, School of Medicine, Southeast University, Nanjing, China; ^2^Nanjing Tongren ENT Hospital, Nanjing, China; ^3^Department of Stomatology, Nanjing Tongren Hospital, School of Medicine, Southeast University, Nanjing, China

**Keywords:** pigment epithelium-derived factor (PEDF), therapeutic prospects, potential, auditory function, signaling pathway, sensorineural hearing loss

## Abstract

The inner ear is a complex and precise auditory perception system responsible for receiving and converting sound signals into neural signals, enabling us to perceive and understand sound. However, the occurrence and development of inner ear diseases and auditory disorders, such as sensorineural hearing loss, remain a global problem. In recent years, there has been increasing research on the treatment of inner ear diseases and auditory regeneration. Among these treatments, pigment epithelium-derived factor (PEDF), as a multifunctional secretory protein, exhibits diverse biological activities and functions through various mechanisms, and has shown potential applications in the inner ear. This minireview comprehensively evaluates the performance of PEDF in sensorineural hearing loss in inner ear and its potential targets and therapeutic prospects.

## 1 Introduction

Inner ear diseases, such as sensorineural hearing loss, inner ear ischemia and anoxia, and deafness can all involve damage to auditory function. Pigment epithelium-derived factor (PEDF), as a protein with cellular protection and anti-angiogenic properties, might offer new avenues for the treatment of inner ear diseases. The promising application of PEDF in the treatment of inner ear diseases has received much research attention. Researchers have attempted to apply PEDF to treat inner ear diseases through approaches such as gene therapy, protein delivery, and drug development. Experimental studies have allowed researchers to speculate that the application of PEDF could reduce the damage to inner ear cells, promote cell survival and regeneration, and improve auditory function. However, the application of PEDF in the treatment of inner ear diseases is still in its early stages, and further research and clinical trials are needed to verify its safety and effectiveness.

PEDF is a member of the serine protease inhibitor (SERPIN) superfamily and exhibits diverse biological activities; although it does not possess protein inhibitory function, hence its rich biological functionality. Initially, PEDF was thought to be a neurotrophic factor present in retinal pigment epithelial cells (Tombran-Tink et al., 1991). However, it was gradually recognized that PEDF is a potent anti-angiogenic factor that can effectively inhibit angiogenesis without affecting the structure and function of normal blood vessels. Additionally, PEDF possesses neurotrophic properties, exhibits anti-tumor effects, reduces oxidative stress, and improves immune function (Bernard et al., 2009).

PEDF is expressed not only in the eyes and brain, but also in many other tissues, such as the blood, liver, kidney, heart, and testis, playing an important role in maintaining and regulating microvascular homeostasis (Brook et al., 2019). Previous study indicated that PEDF is expressed in the inner ear of rats, suggesting its potential expression in the mammalian inner ear (Gleich and Piña, 2008). However, its expression level and pattern in the inner ear have not been confirmed.

The specific effects of PEDF on cochlear hair cells and spiral ganglion cells and its mechanisms are still unclear. Further exploration is needed to uncover the anti-inflammatory and neurotrophic effects of PEDF in the inner ear, as well as its underlying mechanisms.

## 2 The structure of PEDF and its diverse biological activities and functions

PEDF is a 50 kDa endogenous glycoprotein composed of 418 amino acids, encoded by the *SERPINF1* gene, located on human chromosome 17p13 and comprising 12 exons and 34 introns (Becerra et al., 1995). PEDF possesses two important domains: an anti-angiogenic domain composed of 34 amino acids (Asp44-Asn77) and a neurotrophic domain composed of 44 amino acids (Val78-Thr121; Becerra, 2006).

PEDF exhibits anti-angiogenic properties, thereby mitigating inflammatory reactions. Studies have shown that PEDF maintains vascular homeostasis and alleviates inflammation by inhibiting the proliferation and migration of endothelial cells, suppressing angiogenesis, and reducing vascular permeability (Yamagishi et al., 2007; Zhang et al., 2015; Ju et al., 2020). This is particularly important for the inner ear, because normal hearing requires appropriate blood supply and angiogenesis. The anti-angiogenic properties of PEDF help to maintain the balance of blood perfusion in the inner ear, (Gleich et al., 2008; Sheikpranbabu et al., 2009; Eslani et al., 2018) preventing excessive angiogenesis and inflammatory responses that could lead to vascular abnormalities and auditory impairment.

PEDF also exhibits anti-apoptotic and anti-oxidative stress properties. Inner ear cell damage and apoptosis are one of the main causes of hearing loss and inner ear diseases. Studies have found that PEDF can protect inner ear cells from damage and apoptosis through various mechanisms. It regulates cell apoptosis-related signaling pathways, for example, by inhibiting the nuclear factor kappa B (NF-κB) signaling pathway and modulating the expression of B-cell CLL/lymphoma 2 (Bcl-2) family proteins (Zhang et al., 2019; Zhao et al., 2022), thereby alleviating cellular stress responses and promoting cell survival and regeneration.

PEDF is also involved in regulating the differentiation and functional maturation of inner ear cells. Sensory cells and neurons in the inner ear are crucial for normal auditory function. Research has shown that PEDF can promote the survival and development of sensory cells and protect auditory neurons from damage. PEDF plays an important regulatory role in maintaining auditory function in the inner ear through mechanisms such as regulating cell signaling pathways and promoting cell differentiation.

In summary, PEDF inhibits oxidative stress, suppresses inflammatory reactions, has anti-apoptotic effects, promotes cell differentiation, and exerts neuroprotection in the inner ear, making it a potential candidate for the treatment of inner ear diseases and the restoration of auditory function. Researchers have begun to explore the applications of PEDF in the treatment of inner ear diseases and auditory regeneration, with some encouraging results.

## 3 Mechanisms of PEDF’s biological functions

### 3.1 PEDF activates the AKT and Wnt signaling pathways thereby inhibiting oxidative stress reactions

Noise, aging, and ototoxic drugs can cause sensorineural hearing loss via cellular damage caused by oxidative stress reactions (Fujimoto and Yamasoba, 2014; Wu et al., 2022; Baek et al., 2023). Activation of the protein kinase B (AKT) signaling pathway is crucial for the survival of auditory hair cells during ototoxic damage (Jiang et al., 2006) and aging (Sha et al., 2010). In addition, AKT activation leads to activation of downstream mammalian target of rapamycin (mTOR). Studies have shown that upregulation of the phosphatidylinositol-4,5-bisphosphate 3-kinase (PI3K)/AKT signaling pathway contributes to increased hair cell survival rates in ototoxic hearing loss (Bu et al., 2022). Conversely, in noise-exposed mice with hearing loss, levels of PI3K-AKT were reduced (Fan et al., 2023). PEDF plays an important role in protecting retinal pigment epithelial cells from oxidative stress (Kim et al., 2021). In another study, PEDF in olfactory mesenchymal stem cells promoted phosphorylation of the PI3K/AKT/mTOR pathway members to minimize stress reactions after brain injury (He et al., 2021). Therefore, we speculated that sensorineural hearing loss might be reduced through PEDF-mediated activation of the PI3K/AKT/mTOR pathway, thereby decreasing oxidative stress reactions.

Activation of the Wnt (a portmanteau of int. and Wg, standing for “Wingless-related integration site”) pathway has been shown to effectively restore cochlear hair cell-like cell regeneration in mice (Quan et al., 2023; Weng et al., 2023). Conversely, inhibition of the Wnt signaling pathway leads to increased apoptosis, heightened ototoxic damage to spiral ganglion neurons, and worsened hearing loss. The mechanism involves decreased expression of the apoptosis regulator TP53 induced glycolysis regulatory phosphatase (TIGAR) and increased levels of reactive oxygen species (ROS) after inhibition of Wnt/beta-catenin (Liu et al., 2019). Thus, activation of the Wnt pathway plays a crucial role in cochlear hair cell regeneration and spiral ganglion neuron repair. A study has shown that PEDF might reduce oxidative stress through the Wnt signaling pathway (Ma et al., 2017). Therefore, we speculated that PEDF might regulate oxidative stress status through the Wnt signaling pathway in cochlear hair cells or spiral ganglion neurons.

### 3.2 PEDF participates in downregulating inflammatory mediators through the MAPK and NF-κB signaling pathways, and suppresses inflammation

PEDF can improve retinal diseases, such as age-related macular degeneration, by effectively downregulating the mRNA expression of pro-inflammatory cytokines, such as tumor necrosis factor alpha (TNF-α), interleukin (IL)-1β, inducible nitric oxide synthase (iNOS), and IL-17a (Wang et al., 2013). In another study, PEDF reduced the expression of inflammatory cytokines, including monocyte chemoattractant protein-1 (MCP-1) and TNF-α, in retinal extracts, serum, in the culture medium of retinal Müller cells (Filleur et al., 2009).

*In vivo* and *in vitro* evaluation of the anti-inflammatory activity of PEDF in ApoE mice (mice lacking the apolipoprotein E gene), revealed that PEDF significantly decreased the expression of phosphorylated extracellular regulated kinase (ERK)-mitogen-activated kinase (MAPK), p38-MAPK, and JUN N-terminal kinase (JNK)-MAPK. Additionally, overexpression of PEDF resulted in a significant reduction in the expression of inflammatory factors such as IL-1β, IL-6, TNF-α, MCP-1, and matrix metalloprotein-9 (MMP-9; Wen et al., 2017). Thus, there is hope that PEDF might have the potential to reduce inner ear inflammation by lowering the levels of inflammatory mediators.

Researchers have studied the role of the NF-κB signaling pathway in the rat cochlea, particularly its inhibitory effects on inflammation induced by cisplatin toxicity (Kaur et al., 2011). Overexpression of PEDF could restore the activity of the NF-κB pathway and NLR family pyrin domain containing 1 (NLRP1) inflammasomes (Zhao et al., 2022). These observations suggested that PEDF might inhibit inflammatory responses and reduce ototoxic damage in the cochlea.

### 3.3 PEDF might exert anti-apoptotic effects and promote cell survival and differentiation through the MAPK/ERK pathway

The MAPK/ERK pathway plays a vital role in maintaining cell survival and protecting spiral ganglion neurons from apoptosis, demonstrating neuroprotective effects (Lallemend et al., 2003). *In vitro*, PEDF has been shown to activate the MAPK/ERK pathway, thereby promoting the migration and invasion of tumor cells (Chen et al., 2021). Another study reported that the MAPK signaling pathway is involved in the activation of JNK, which can inhibit the cell apoptosis caused by noise-induced hearing loss (Hu et al., 2009). This suggested that PEDF might play a role in protecting hearing through the MAPK/ERK and JNK pathways.

### 3.4 PEDF has the potential to activate anti-apoptotic pathways and achieve neuroprotective effects

PEDF has the potential to promote neuronal differentiation, regeneration, and the survival and differentiation of stem cells, making it a promising candidate for regenerative therapies (Ramírez-Castillejo et al., 2006; Brook et al., 2020). In a study on traumatic brain injury, elevated levels of PEDF were observed, indicating a potential activation of its neuroprotective functions through the inflammatory response and neural proliferation after injury (Terzi et al., 2015). Emerging evidence suggests that PEDF might play a role in protecting neurons in neurodegenerative diseases. A recent analysis identified PEDF as a promising therapeutic agent for multiple sclerosis, based on its ability to enhance remyelination by increasing the number of oligodendrocyte precursor cells and mature oligodendrocytes (Hooijmans et al., 2019).

PEDF appears to mediate the neuroprotective effect on retinal progenitor cells by binding to its receptor, PEDF-R, and activating anti-apoptotic pathways, such as Bcl-2, and blocking the translocation of apoptosis-inducing factor (AIF) and photoreceptor cell death (Subramanian et al., 2013). The biological functions of PEDF might not operate independently, but rather through the interplay and intersection of multiple functions. Moreover, the neuroprotective effect might be based on its anti-apoptotic function.

Overall, PEDF exhibits various mechanisms in achieving its biological functions, including reducing oxidative stress reactions, suppressing inflammation, promoting cell survival and differentiation, and achieving neuroprotective functions through signaling pathways such as AKT, Wnt, MAPK/ERK, and NF-κB.

## 4 Application prospect and conclusions

In summary, PEDF has received widespread attention for its potential applications in the inner ear. PEDF is considered a promising therapeutic agent to treat inner ear diseases. Although inner ear diseases often result in hearing loss, PEDF’s ability to alleviate cellular stress and inflammatory responses, as well as promote cell survival and regeneration, make it a beneficial option for treatment. PEDF’s potential as an intervention target for oxidative stress regulation and neurotrophic intervention in some inner ear diseases has theoretical support in clinical applications. Experimental results have shown that the application of PEDF can alleviate cellular damage in the inner ear, and promote cell survival and regeneration, thereby improving auditory function. However, the specific mechanisms by which PEDF activates auditory hair cells and spiral ganglion cells in the treatment of inner ear diseases are still mostly unknown, and further research and clinical trials are needed to verify its safety and efficacy.

## Author contributions

ZS: Writing – original draft, Conceptualization, Formal analysis, Resources. XL: Data curation, Investigation, Writing – original draft. GL: Investigation, Methodology, Supervision, Writing – original draft. YX: Formal analysis, Project administration, Writing – original draft. JM: Supervision, Validation, Writing – review & editing. WM: Funding acquisition, Project administration, Writing – review & editing. SH: Writing – review & editing, Supervision, Validation.
